# Impact of offensive-reward rules on young basketball players’ performance during small-sided games

**DOI:** 10.1371/journal.pone.0313656

**Published:** 2025-01-03

**Authors:** Eduardo Abade, Bruno Figueira, Diogo Coutinho, Hugo Folgado, Catarina Moreira, Bruno Gonçalves

**Affiliations:** 1 Research Center in Sports Sciences, Health Sciences and Human Development, CIDESD, University of Trás-os-Montes e Alto Douro, UTAD, Vila Real, Portugal; 2 Department of Sports Sciences, Exercise and Health, University of Trás-os-Montes and Alto Douro, UTAD, Vila Real, Portugal; 3 Portugal Football School, Portuguese Football Federation, Oeiras, Portugal; 4 Departamento de Desporto e Saúde, Escola de Saúde e Desenvolvimento Humano, Universidade de Évora, Évora, Portugal; 5 Comprehensive Health Research Centre (CHRC), Universidade de Évora, Évora, Portugal; 6 Research Center in Sports Sciences, Health Sciences and Human Development, CIDESD, University of Maia, UMAIA, Maia, Portugal; University of Castilla-La Mancha, SPAIN

## Abstract

This study aimed to investigate the impact of different offensive-reward-related rules on the physical performance, perceived exertion and enjoyment of young basketball players during small-sided games (SSG). Eighteen youth male players (age: 13.3±0.9y; height: 167.0±13.1cm; weight: 50.6±11.5kg; years of practice: 3.1±1.2y) participated in three distinct 3x3 SSG (14x15m pitch) experimental conditions: i) 3x3NORMAL, regular 3x3 game; ii) 3x3POINT-POSS, where the team in possession was rewarded a new ball possession after scoring; iii) 3x3PASSES, where the score was determined by the number of passes made before converting a basket. Players’ performance was measured using global positioning systems, rating of perceived exertion and the Physical Activity Enjoyment Scale. Players covered more distance for total (*p*< .001), low (*p*< .01) and moderate-speed distance (*p*< .05), and presented a higher game pace (*p*< .001) during the 3x3POINT-POSS than in the other conditions. A higher number of accelerations (1–1.99 m·s^-2^, *p* < .01) and decelerations (>2 m·s^-2^, *p* < .01) were also reported in the 3x3POINT-POSS. Despite the higher external load in the 3x3POINT-POSS, players reported higher perceived exertion during the 3x3PASSES (small to moderate effects). In addition, players reported the 3x3POINT-POSS as being more enjoyfull than the 3x3NORMAL. In conclusion, coaches may consider incorporating the 3x3POINT-POSS to enhance players’ external load while simultaneously increasing enjoyment and decreasing perceived exertion. Conversely, the 3x3PASSES condition may be useful for promoting adaptive behaviors under a higher perception of effort. Thus, coaches should carefully choose the type of rewards when designing training tasks for youth basketball players.

## Introduction

Team sports are characterized by a dynamic interplay of cooperation and confrontation between opposing teams [1]. In possession, the team strives to exhibit coordinated behaviors to conquer space collectively and advance towards specific objectives. Simultaneously, without possession, players employ individual, group, and collective strategies to impede the progress of their opponents [2, 3]. The proficiency of players in executing such coordinated movements hinges on their capacity to recognize and use pertinent information from the surrounding environment [4]. For instance, a player without possession who, during an offensive set play, adeptly scans the environment, strategically position himself to receive the ball, and factors in considerations such as the proximity to the player in possession, distance to the nearest defender, positioning of remaining defenders [5], and targe distance / location [6] significantly enhances the team’s chances of scoring. Therefore, a player’s ability to perceive and maneuver within dynamic and unpredictable environments is crucial for achieving successful performances [7], specially in team sports played under diverse environmental conditions that demand intricate decision-making in high-pressure situations [8].

Basketball exemplifies a team sport where the players’ capacity to base their actions on environmental information is determinant for achieving successful performances [9, 10]. Consequently, basketball is distinguished by its strategic intricacies, demanding teams to deploy an array of standardized offensive strategies in conjunction with complex defensive tactics [11]. In fact, basketball offensive moment demands consistent and nuanced tactical modifications [12]. Therefore, players are compelled to collaboratively refine their behaviors, adapting to the inherent flux and variations intrinsic to the dynamic nature of the game [2, 3]. In addition to the critical role of players’ decision-making, their physical [13] and technical attributes [14] may serve to either limit or enhance their opportunities for action [15]. Thus, this fusion of strategy and adaptability positions basketball not only as a physical pursuit but also as a cerebral endeavor, necessitating a seamless integration of athleticism and strategic foresight [16]. Consequently, training tasks should not only accentuate players’ decision-making prowess but also mold their physical and technical capacities.

To assist coaches in the design of enriching training environments, sports science researchers have explored diverse training methodologies aimed at developing players’ decision-making and adaptability [17–19]. An exemplar among these is the constraint-led approach (CLA), which underscores the utilization of task-specific constraints to stimulate players in exploring varied movement solutions and refining their tactical behavior [20, 21], while providing and adequate physical stimulous. Although these variations can be incorporated into real or simulated game practices, such as 5v5 in basketball [22], CLA often finds application in small-sided games (SSG), which consists in game-based tasks that entails modifications compared to official competitive games [18]. The underlying premise is that players’ movement behavior is a product of their ability to identify and utilize information for goal-directed actions [23]. Consequently, different task boundary conditions inevitably alter the available information [18], and subsequently guiding players towards distinct movement behaviors, eliciting different physical demands [21]. Conscious of these impact, research has been exploring how different task-related boundary conditions affects players’ performance. For example, literature has shown that decreasing the number of players (e.g., 2x2) may increase the technical and physical demands of training practices compared to larger formats (e.g., 4x4) [24]. More recently, it has been demonstrated that coaches can emphasize individual behaviors by increasing the number of targets from 2 to 4, concurrently elevating the external load due to higher team dispersion [21]. These findings emphasize the profound influence that changing these constraints can have on enhancing players’ skills and adaptability.

Through the strategic manipulation of these constraints, coaches and researchers can craft training environments that encourage players to refine their decision-making, adaptability and physical capacities. In fact, changes in technical and/or tactical behavior as a result of different game based formats may result in distinct physical and physiological adaptations [25]. For example, reducing effective playing area may decrease players’ physical responses [26]. Also, changing rules to promote defensive pressure may increase acceleration (ACC) demands and heart rate responses [27]. Despite the increasing volume of research in this domain over recent years, the majority has focused on adult players [28, 29] or youth players in later developmental stages [13, 21, 22]. Consequently, a recent literature review has underscored the necessity for more studies encompassing players’ performance with various task manipulations, particularly in younger age groups (U10 to U14), to support the holistic development of short and long-term goals [30].

While there is no existing literature specifically exploring possession or reward rules, some research has delved into the manipulation of the number of players and rules. A study compared youth players (U14 and U15 age groups) performance across different game conditions, including a 3x3 regular SSG, 4x3 numerical superiority, 3x3 defensive pressure (i.e., close man-marking), and a 3x3 close shoot (i.e., points only allowed inside the lane area) [27]. The findings indicated enhanced offensive performance in the numerical superiority condition, whereas defensive pressure was shown to emphasize physical performance. Conversely, the close shoot condition resulted in lower offensive performance, possibly due to players adopting more group-defensive approaches near the target [27]. The authors postulated that certain rules might limit players’ performance (e.g., close shoot), highlighting the need for further research exploring how different rules affect youth players’ behaviour [27, 30]. From this point-of-view, no study to date has addressed how reward rules may affect players’ performance. Rewards seems to affect players’ decision-making in team sports [31], positively encourage players’ to move [32] and enhance learning in human subjects performing implicit motor-learning tasks [33]. On the other hand, restricted constraints that reduce the number of opportunities for action may limit the degree of freedom and have a negative transfer to players behaviour [34].

Both reward and punishment may motivate human behaviour, although it is not clear exactly how they affect skill performance [35]. The current study seeks to address this gap by investigating how different offensive-reward rules impact young basketball players’ physical exertion, perceived exertion, and enjoyment during 3x3 SSG.

## Materials and methods

### Participants

Power analysis was performed to determine the required sample size. The sample size was calculated with G*Power (Version 3.1.9.6. Institut für Experimentelle Psychologie, Düsseldorf, Germany) for an effect size of 0.7, an α of 0.05, and a power of 0.8 (1–β) [36]. The total sample sized computed with this method was a minimum of 15 players. A previous study using a similar age group category used 18 players to test them during SSG [37], thus, in line with this protocol a total of eighteen youth trained [38] male basketball players (age: 13.3±0.9y; height: 167.0±13.1cm; weight: 50.6±11.5kg; years of practice: 3.1±1.2y) participated in this study. The participants had regular training sessions every week, three times a week for 90 min and one game at the weekend at regional and national competitions. While the research group has stablished failing to any session or lower limb injury as exclusion criteria [39], none of the players had any factors that could hinder or influence any of the requested tests. Written informed consent was obtained from each guardian, as all the players were minors. After reading and explaining the tests, the guardians agreed to allow their wards to participate in the tests (between 1^st^ and 31th may, 2023). The project and protocol were approved by the Ethics Committee and Academic Services of the University of Évora (code 21074). It was conducted in accordance with the Helsinki Declaration considering ethical principles for medical research involving human subjects.

### Design

All players were tested during three sessions over three weeks (i.e. one session per week) during the middle of the in-season competitive period (between the 1^st^ to 31th of May, 2023). The initial session, conducted in the first week, served as a familiarization session. This allowed participants the opportunity to engage in the protocol for the first time, providing them with a chance to adapt and become acquainted with the rules of the study. Then, the remaining sessions were used to test three experimental conditions (3x3NORMAL, 3x3POINT-POSS 3x3PASSES). In total, condition was tested three times, one per session. The session started at the same time of the day, to avoid the effects of circadian rhythms on the results, and over similar weather conditions. Each session lasted 34 minutes, consisting of a 10-minute warm-up followed by a 24-minute half-court basketball SSG. Subjects performed the same standardized warm-up consisting of moderate-intensity jogging (4 minutes), static and dynamic stretching (4 minutes), and accelerative running bouts (2 minutes). Data collected during the warm-up periods were not included for analysis.

### Procedures

For each testing day, the head coach selected eighteen players that allowed to divide the group into 6 balanced teams (three groups of two teams, 3x3 games), considering the coach perception of the players physical, technical, tactical, and perceptual skills. Throughout the testing sessions, the competing teams remained consistent, ensuring stability, while the players were randomly exposed to all three experimental conditions on different days to enhance data generalization. In each day players performed three bouts of 6-min interspersed with a 3-min passive recovery in between, where each bout consisted in one of the three experimental conditions: (i) 3x3NORMAL game consisted in playing a 3x3 standard game; (ii) 3x3POINT-POSS game followed the same rules, except that if the attacking team scored a basket, team retained possession, and the game resumed with a check ball from the top of the arch at the 3-point line; (iii) 3x3PASSES game, where instead of counting 1 or 2 points for a made basket, the score was determined by the number of passes made before the basket was converted during the possession. The order of the experimental conditions on the testing days was randomly assigned. The 3x3 SSG were conducted on a 14x15m outdoor half-court pitch, adhering to the 3x3 International Basketball Federation (FIBA) rules [28]. No coach feedback or encouragement was allowed during the conditions.

### Data collection

Activity demands were measured using wearable 18-Hz GPS units (STATSports Apex, Northern Ireland, Newry). The accuracy of this device has been previously examined, reporting a nearly perfect criterion validity to measure distance during team sport specific movements (ICC = 0.98) [40]. All devices were activated 15-min before the data collection to allow acquisition of satellite signals in accordance with the manufacturer’s instructions. Data were subsequently downloaded and processed using corporate software (STATSports Apex, Northern Ireland, Newry). The following categorization was used to indicate different activity intensities based on previous basketball research [41, 42]: low-speed (<6km·h^-1^); moderate-speed (6.01—12km·h^-1^); high-speed (12.01—18km·h^-1^); and maximal-speed (>18 km·h^-1^). ACC and deceleration (DEC) intensities was classified as low (0.5–0.99m·s^-2^); medium (1–1.99m·s^-2^); and high (>2m·s^-2^). Time elapsed since the last ACC and DEC was also computed.

Each player gave an individualized rating of perceived exertion (RPE) and Physical Activity Enjoyment Scale (PACES) rates immediately after each game. RPE was carried using Borg’s 10-point Likert scale (with “1” indicating a minimum response and “10” indicating a maximum response). The PACES Scale allowed to assess the enjoyment of each participant after completing each activity [43]. It was used a 3-item scale that begins with the statement "When I am physically active…", with each item rated on a 5-point Likert scale ranging from 1 ("strongly disagree") to 5 ("strongly agree"). These three items were "It’s a lot of fun", "It’s very gratifying" and "It’s very stimulating".

### Statistical analysis

After preliminary inspections for distribution and normality, repeated measures analysis of variance was processed to identify the effect of the game condition (3x3NORMAL, 3x3POINT-POSS and 3x3PASSES) on the considered variables. The results are shown in mean ± standard deviation (M±SD). Pairwise differences were assessed with Bonferroni post hoc. The statistical analysis was performed using the Statistical Package for the Social Sciences software (SPSS, Inc., Chicago, IL, USA), and statistical significance was set at *p*< .05.

An estimation techniques approach was carried to overcome the shortcomings associated with traditional N-P null hypothesis significance testing [44, 45]. The Cohen’s *d*_*unbiased*_ (*d*_*unb*_) with 95% confidence intervals (CI) as effect size (ES) (an unbiased estimate has a sampling distribution whose mean equals the population parameter being estimated) was applied to identify pairwise differences [44]. Thresholds for ES statistics were: 0.2, 0.5, and 0.8 for small, medium, and large [46].

## Results

The descriptive and inferential results of the effect of game condition on the independent variables is presented in Table 1.

**Table 1:** Descriptive (mean±SD) and inferential analysis (repeated measures).

Variables	Game Conditions			F	*p*	η^2^_p_
	3x3NORMAL	3x3POINT-POSS	3x3PASSES			
Total distance covered (m)	364.3±94.1 ^a^	404.8±69.5 ^c^	369.6±73.6	8.62	< .001**	.337
Low-speed (0–6 km·h^-1^)	217.3±27.6 ^a^	230.7±15.6 ^c^	217.7±29.2	6.09	.006**	.264
Moderate-speed (6–12 km·h^-1^)	133.8±64.2 ^a^	159.2±61.8	140.8±54.5	4.38	.020*	.205
High-speed (12–18 km·h^-1^)	12.9±14.5	14.8±13.7	11.1±12.8	1.28	.291	.070
Maximum-speed (>18 km·h^-1^)	0.1±0.3	0±0	0±0	2.13	.135	.111
Average speed (km·h^-1^)	3.6±0.9 ^a^	4.1±0.7 ^c^	3.7±0.7	8.59	< .001**	.336
Accelerations (n)						
0.50–0.99 m·s^-2^	22.6±4.6 ^b^	21.3±3.8 ^c^	18.6±4.9	5.23	.010*	.235
1–1.99 m·s^-2^	26.4±5.3 ^a^	32.7±6.7	29.9±8.6	6.29	.005**	.27
>2 m·s^-2^	19.9±10.0	22.6±8.1	18.7±7.8	2.26	.120	.117
Time elapsed since the last acceleration (s)	198.7±204.7	86.7±76.0 ^c^	334.3±284.3	7.08	.003*	.294
Decelerations (n)						
0.50–0.99 m·s^-2^	21.5±6.4	21.3±4.8	20.3±5.8	0.372	.692	.021
1–1.99 m·s^-2^	31.3±6.9	34.4±6.6	32.4±7.5	1.49	.240	.081
>2 m·s^-2^	15.9±7.4 ^a^	20.8±8.1 ^c^	16.7±7.2	5.41	.009**	.241
Time elapsed since the last decelerations (s)	268.6±215.8 ^a^	53.6±43.8 ^c^	116.1±267.9	8.41	.001**	.331
Rate of perceived exertion (0–10)	2.4±1.3 ^b^	2.9±1.5	3.3±1.7	6.4	.004**	.274
Physical Activity Enjoyment Scale						
"It’s a lot of fun", (1–5)	3.9± 0.8	4.3± 0.7	4.2±0.7	2.66	.084	.135
“It’s very gratifying” (1–5)	3.5±0.9	3.7±0.8	3.9±0.9	2.19	.127	.114
“It’s very stimulating” (1–5)	3.9±0.8	3.8±0.8	4.0±0.7	1.1	.345	.061

*p < .05

**p < .01

Post-hoc differences are identified as

^a^3x3NORMAL vs 3x3POINT-POSS

^b^3x3NORMAL vs 3x3PASSES

^c^3x3POINT-POSS vs 3x3PASSES.

**Fig 1:**
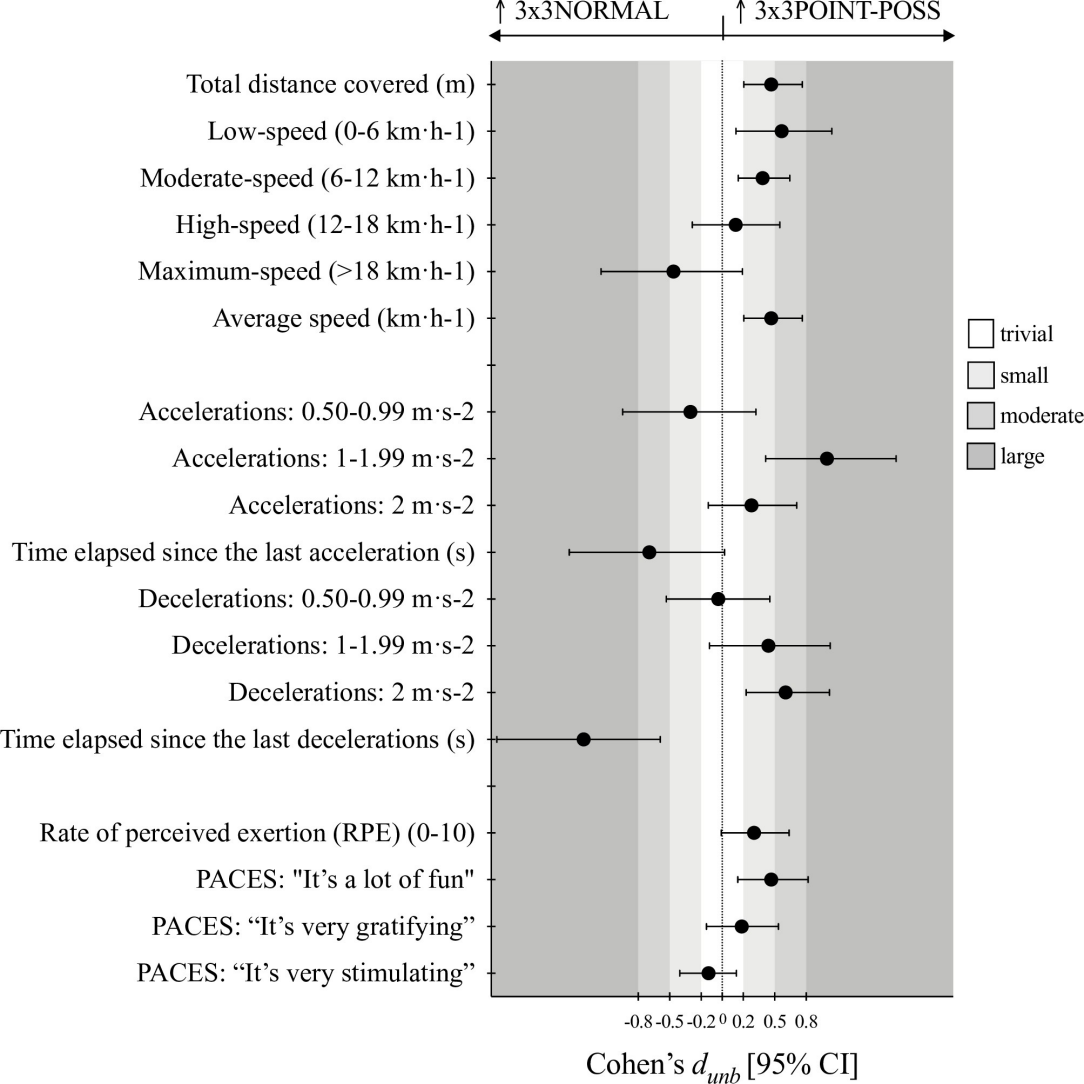
Cohen’s *d*_*unbiased*_ results for the pairwise comparisons of the 3x3NORMAL vs. 3x3POINT-POSS game conditions.

**Fig 2:**
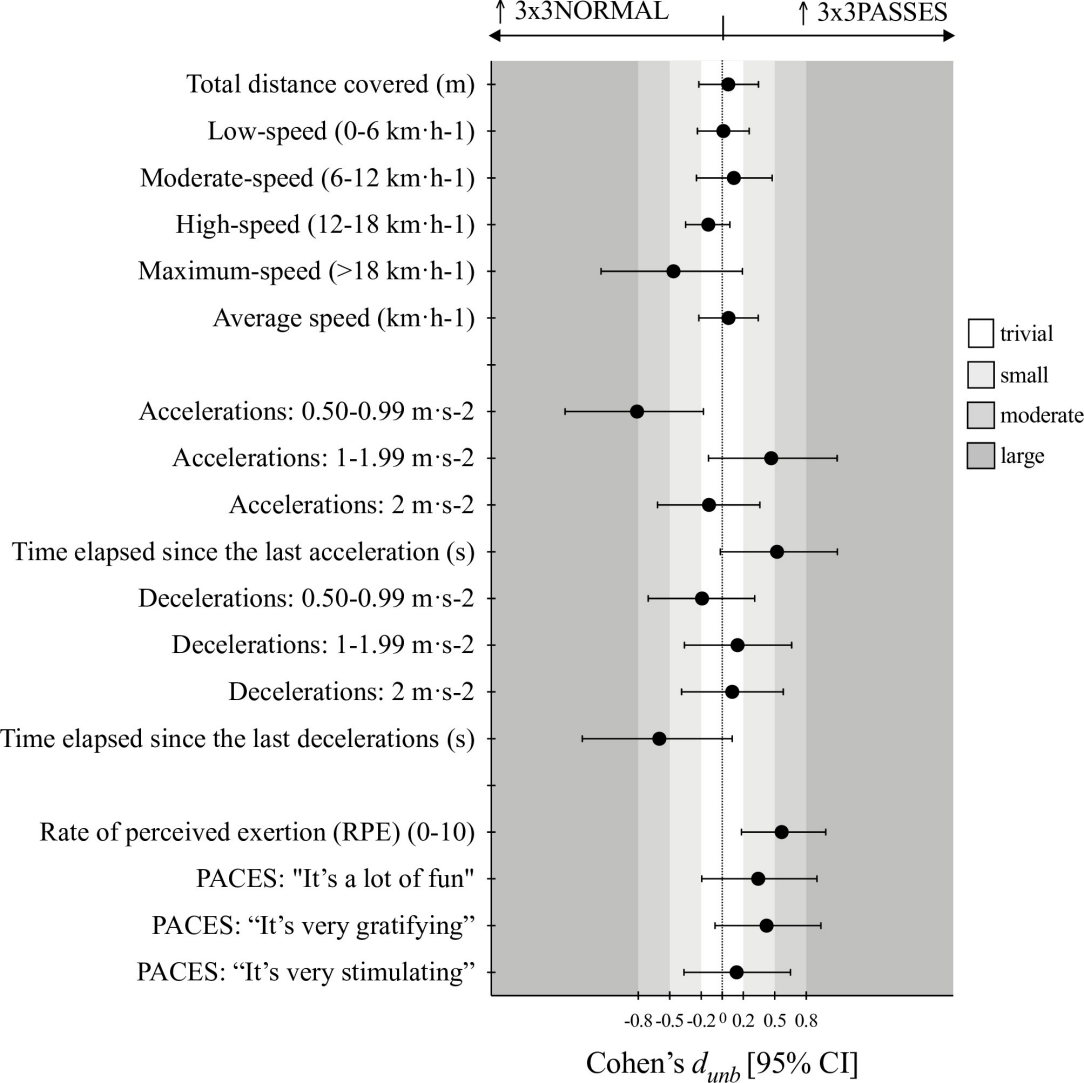
Cohen’s *d*_*unbiased*_ results for the pairwise comparisons of the 3x3NORMAL vs. 3x3PASSES game conditions.

**Fig 3:**
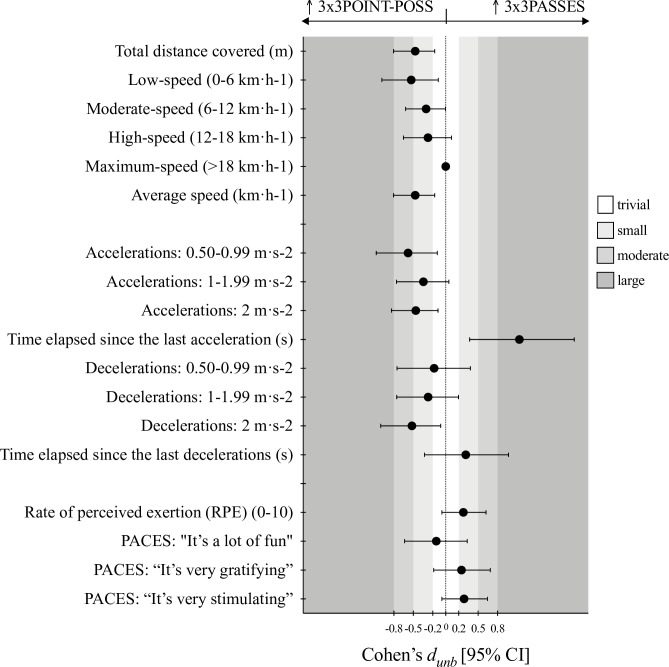
Cohen’s *d*_*unbiased*_ results for the pairwise comparisons of the 3x3POINT-POSS vs. 3x3PASSES game conditions.

Figs 1–3 shows the ES expressed as *d*_*unb*_ for the pairwise differences, 3x3NORMAL vs. 3x3POINT-POSS, 3x3NORMAL vs. 3x3PASSES, and 3x3POINT-POSS vs. 3x3PASSES, respectively.

It was observed that in the 3x3POINT-POSS condition, players achieved significant higher values in the distances covered, specifically in the total distance (F = 8.62, *p*< .001, η^2^_p_ = .337) and in distance covered at both low- (F = 6.09, *p*< .006, η^2^_p_ = .264) and moderate-speed (F = 4.38, *p*< .020, η^2^_p_ = .205). The *d*_*unb*_ ranged from small to moderate in the pairwise comparisons with 3x3NORMAL and 3x3PASSES. Also, the average speed of the players movement during game condition was higher for 3x3POINT-POSS (F = 8.59, *p*< .001, η^2^_p_ = .336) with small ES.

Regarding to the ACC and DEC variables, significant differences were identified for the for the number of medium intensity ACC (F = 6.29, *p* < .005, η^2^p = .270), time elapsed since the last ACC (F = 7.08, *p* = .003, η^2^p = .294), number of high intensity DEC (F = 5.41, *p* = .009, η^2^p = .241), and time elapsed since the last DEC (F = 8.41, *p* = .001, η^2^p = .331). Overall, the 3x3POINT-POSS game condition exposed players to higher number of actions, from small to large *d*_*unb*_, as well as drastically decreased de time elapsed since the last ACC/ DEC when comparing to the other conditions.

No significant differences were identified for the RPE and physical activity enjoyment scales (PACES). However, clear trends were depicted in the *d*_*unb*_ results. The 3x3PASSES game condition shown small to moderate higher effort perception and players considered this game as well as the 3x3POINT-POSS more fun and gratifying then playing the 3x3NORMAL game.

## Discussion

The main findings of this study revealed that the 3x3POINT-POSS condition imposed the greatest physical demands on the players. This condition was also viewed as more enjoyable and rewarding, especially when contrasted with the 3x3NORMAL. On the other hand, the 3x3PASSES condition was perceived to necessitate a higher level of effort.

The 3x3POINT-POSS elicited higher total distance covered, distance at low to moderate-speeds, while also higher game pace. This condition allowed players the opportunity to earn a new ball possession upon scoring, potentially motivating teams to exert more effort in attempting to score and retain possession for subsequent shots. In fact, the players reported finding this condition more enjoyable and rewarding. Sports psychology research suggests that rewards can positively impact physical performance in team sports, fostering increased cohesion and effort among players [32]. Indeed, the concept of reward, fundamental in influencing human behavior and decision-making in team sports, is echoed in this context [31]. Consequently, players may intensify the game pace and cover more distance in their attempts to score successfully, thus securing a new ball possession.

While being rewarded with a new ball possession for the offensive team during the 3x3POINT-POSS, the defensive team also appears to intensify their efforts and pace to exert pressure on the opponents. This is evident in the ACC profiles, indicating an increase in the number of ACC (1–1.99m·s^-2^) and DEC (>2m·s^-2^), along with a shorter time elapsed since the last ACC / DEC. These findings align with existing research, suggesting that higher ACC profiles often occur during basketball defensive moments [47]. Such defensive adjustments are likely related to continuous spatial positioning based on the ball’s location and the movements of opposing players [2, 3, 9]. Supporting this observation, a prior study demonstrated an increased number of defensive actions in a 3x3 high press game compared to a 3x3 regular game. This heightened defensive activity stemmed from players attempting to closely mark their direct opponents to limit their progression opportunities [27]. In addition, Bredt et al. [27] found a higher volume of ACC in the 3x3 high press condition, indicative of increased defensive actions. While speculative, it is plausible that players adopted a high-press defensive strategy during the 3x3POINT-POSS, aiming to recover ball possession, thereby contributing to the elevated number of ACC and DEC.

Overall, the 3x3POINT-POSS was reported as the most physically demanding task. Despite that, it reported the lowest RPE values, which may suggest an intriguing interplay between physical exertion and perceived effort, potentially influenced by motivational factors [48]. This aligns with the psychobiological model of endurance performance, which posits motivation as a critical factor in modulating perceived effort [49]. The athletes’ perception of the 3x3POINT-POSS as enjoyable and stimulating implies that motivation may have played a role in reducing their perception of effort during the exercise. This factor seems to being line with the concept of effort perception being influenced by motivation [50]. In turn, the 3x3PASSES was perceived as demanding higher effort by the players. In this condition, the players were awared by the number of passes performed, which may increase the time spent in the offensive process. In fact, this condition (i.e., 3x3PASSES) may serve as a valuable tool to encourage players to enhance their ability to use passing as a strategic offensive approach. Accordingly, a previous study showed that players perceive the task as more demanding when being exposed to offensive roles (i.e., when only one team attacks continuously) compared to defensive roles [28]. Moreover, defender’s perception of effort should be considered due to need of avoiding opponents from shooting or circulating the ball. Conversely, the 3x3NORMAL condition involved a ball possession exchange upon scoring, necessitating players from both teams to adjust their spatial occupation in preparation for upcoming actions, leading to lower physical demands.

This study provides valuable insights to assist coaches in tailoring relevant training tasks for youth basketball players. However, certain limitations should be acknowledged. Primarily, the focus of this study was on the effects of different offensive-reward rules on players’ physical performance, perceived exertion, and enjoyment. While these variables contribute significant value to the practical application, a more comprehensive understanding of players’ response to such rules would involve the inclusion of technical, tactical, and positional variables. Additionally, it is noteworthy that players from different age groups may exhibit varying adjustments to the same task boundary conditions manipulations. Future research should address this limitation while also considering the incorporation of defensive-reward rules.

## Conclusions and practical applications

This study investigated the impact of different offensive-reward rules on players’ physical performance, perceived exertion and enjoyment during 3x3SSG. Notably, the 3x3POINT-POSS condition induced higher physical demands compared to other conditions. The incentive of being rewarded with a new ball possession upon scoring heightened motivation, as reflected in the PACES questionnaire. Interestingly, despite the higher external load reported during 3x3POINT-POSS, players perceived the 3x3PASSES condition as more demanding. This perception may be attributed to the belief that offensive tactical missions are generally more challenging. These findings have important practical implications for coaches. By understanding how reward systems influence player performance and perceptions, coaches can tailor training drills to enhance physical, tactical, and technical skills. For teams favoring fast-paced, high-pressure gameplay, the 3x3POINT-POSS drills may boost motivation and maintain player engagement. Conversely, the 3x3PASSES condition may be used as a valuable tool to encourage players to enhance their ability to use passing as a strategic offensive approach under a higher perception of effort.
